# Coronary Slow-Flow Phenomenon as an Underrecognized and Treatable Source of Chest Pain: Case Series and Literature Review

**DOI:** 10.1177/2324709618789194

**Published:** 2018-07-17

**Authors:** Chikezie Alvarez, Henry Siu

**Affiliations:** 1 St. Francis Medical Center, Trenton, NJ, USA; 2 Thomas Jefferson University, Philadelphia, PA, USA

**Keywords:** coronary slow-flow, coronary angiography, chest pain, noninvasive stress test, calcium channel blocker, TIMI frame count

## Abstract

*Background*. Coronary slow-flow phenomenon (CSFP) is characterized by delayed distal vessel opacification of contrast, in the absence of significant epicardial coronary stenosis. CSFP has been reported as a cause of chest pain and abnormal noninvasive ischemic tests and is often underrecognized. *Material and Methods*. Charts and angiographic records from our institution were reviewed to identify 15 consecutive patients who were diagnosed with CSFP from January 2016 to January 2017. *Results*. Of the 15 patients (4 females and 11 males) studied, the mean age was 59.1 years (range = 45-86 years); all had left ventricular ejection fraction >45% and without significant valvular stenosis/regurgitation. The indication for coronary angiography for all 15 patients was chest pain with abnormal noninvasive tests. Of the 11 patients who underwent previous coronary angiograms, all revealed prior evidence of CSFP. None of these patients were on calcium channel blockers (CCBs) or long-acting nitroglycerin agents before angiography. Intracoronary CCBs were effectively utilized to alleviate the angiographic finding (improvement in Thrombolysis in Myocardial Infarction frame count) in all 15 patients. Oral CCBs were started with subsequent improvement in all 15 patients (mean follow-up time = 13.6 months). *Conclusion*. Coronary slow-flow should be a diagnostic consideration in patients presenting with chest pain and abnormal noninvasive ischemic testing with nonobstructive epicardial vessels. CSFP remains underrecognized, and the specific standard of care for treatment has not been established. In each of the 15 cases, intracoronary nifedipine resolved the angiographic manifestation of coronary slow-flow. Furthermore, in follow-up, all patients improved symptomatically from their chest pain after oral CCBs were initiated.

## Introduction

Coronary slow-flow phenomenon (CSFP), also known as cardiac syndrome Y, is characterized angiographically by delayed distal vessel opacification in the absence of obstructive coronary artery disease and represents a pathology related to underlying dysfunction of microvascular resistance.^1^ The diagnosis of CSFP is made via coronary angiography based on either a reduced Thrombolysis in Myocardial Infarction (TIMI) flow grade of 2 or increased corrected TIMI frame count of greater than 27 frames in one or more epicardial vessel.^2,3^

The prevalence of CSFP has been reported to range between 1% and 5% of diagnostic coronary angiograms and is classically described in young male smokers with recurrent chest pain.^2,4,5^ Regarding the coronary vasculature, the left anterior descending (LAD) artery, even when corrected for length, is most often involved (50% to 90% of the time), followed by the right coronary artery (28% to 45%) and the left circumflex (-20%).^6,7^ Coronary angiograms in patients with CSFP are often referred to as “normal” or “mild nonobstructive disease,” which lends itself into classifying these phenotypical patients as having “chest pain with a negative cardiac catheterization.” Perhaps due to the lack of a fully understood pathophysiology, CSFP is frequently not identified as a root cause of abnormal ischemic testing and recurrent chest pain symptoms.

Various medications have been evaluated for the treatment of CSFP. However, the actual efficacies of the majority of these pharmacological agents have not been established. Oral calcium channel blockers (CCBs) can attenuate the microvascular effects associated with coronary slow-flow.^8^ Studies have utilized intracoronary (IC) CCBs to improve the TIMI frame count in patients with CSFP on catheterization. To our knowledge, however, no previous studies have uniformly evaluated the subsequent use of oral CCBs in patients whose angiographic slow-flow resolved with IC CCBs.^9-11^

We, therefore, reviewed 15 consecutive patients who were diagnosed with CSFP via the TIMI frame count method after IC administration of nifedipine. Our study focuses on the role of CCBs in alleviating this angiographic condition as well as its potential applicability for symptomatic treatment.

## Material and Methods

**Figure 1.**
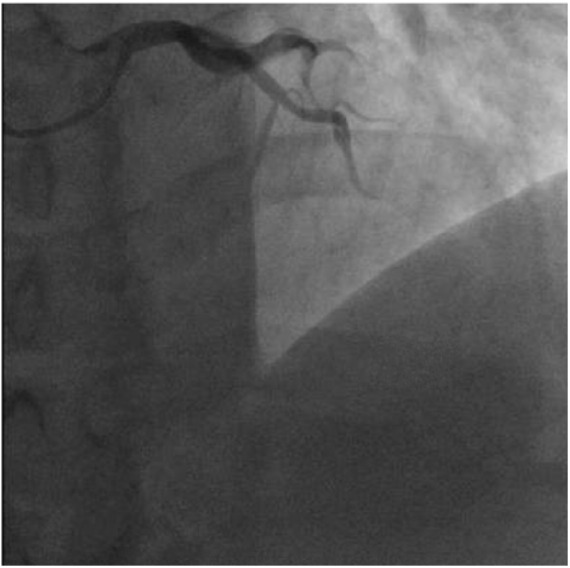
Coronary slow-flow in both the left anterior descending (LAD) and left circumflex (LCX). Coronary angiogram at the 25th cine frame (utilizing 30 frames per second acquisition) revealing contrast opacification only up to the mid-vessel segment of the LAD and LCX.

**Figure 2. fig2-2324709618789194:**
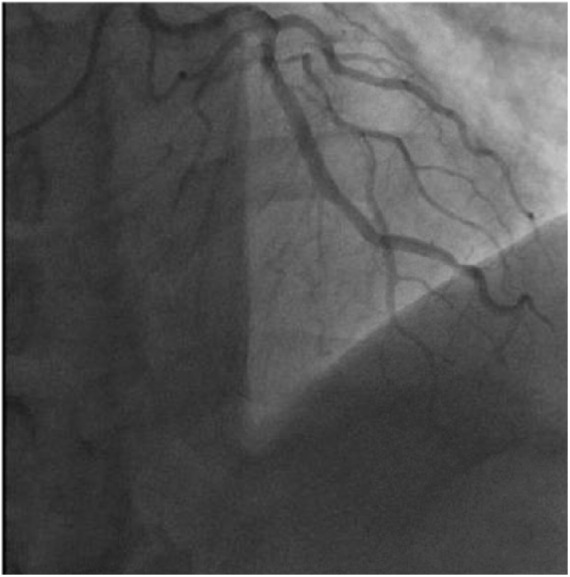
It took 110 frames for the contrast to reach the distal vessel segment of the left anterior descending and left circumflex; significant contrast “washout” is noted with delayed or “sluggish” contrast filling.

Charts and angiographic records from our institution were reviewed of 15 consecutive patients who were diagnosed with CSFP from January 2016 to January 2017. Angiograms were evaluated and reviewed, and TIMI frame counts were verified. Slow-flow was defined by a frame count greater than 27 for all vessels (for the LAD, the frame count was divided by 1.7 to correct for the longer vessel length), which was a definition adapted from the previous methodology from Gibson et al.^3^ Coronary angiograms were performed using power contrast injection Medrad Avanta, utilizing a standard flow rate of 4 mL/s, volume of 4 mL, and pressure limit of 450 PSI (pounds per square inch). Of note, cine fluoroscopy in our institution was acquired at 15 frames per second, and therefore, the recorded frame count was multiplied by 2. Furthermore, all subjects had TIMI-2 flow, which is defined by ≥3 beats to opacify prespecified branch points in the distal vasculature.^2^ Eleven of the 15 patients had previous angiograms (spanning 2008-2014), which were obtained and reviewed. Four of the 15 patients never had previous coronary angiography (see Figures 1 [Fig fig2-2324709618789194]-3).

**Figure 3.**
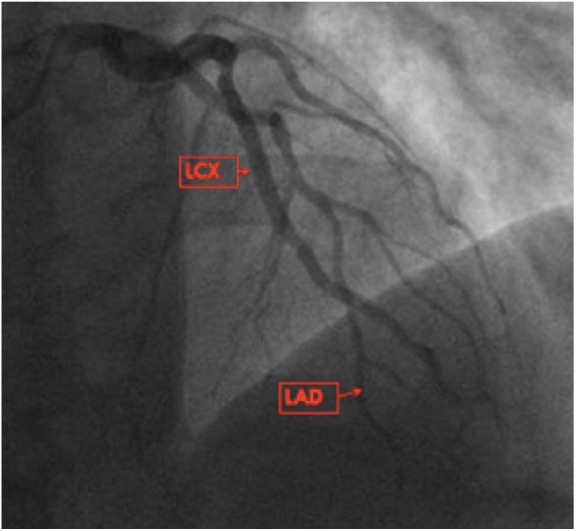
Coronary angiogram after administration of intracoronary nicardipine, brisk vessel opacification by the 25th cine frame is noted, indicating resolution of coronary slow-flow.

## Results

The diagnosis of CSFP was established in 15 patients by an initial corrected TIMI frame count >27 frames (mean of 105 frames in vessels affected by CSFP), with improvement to <27 frames after administration of IC nifedipine (average dose = 200 µg). Of the 15 patients (4 females and 11 males) studied, the mean age was 59.1 years (range = 45-86 years; Table 1); all had left ventricular ejection fraction >45% (Table 2). The indication for coronary angiography for all 15 patients was chest pain with abnormal noninvasive tests; 11 pharmacologic nuclear stress tests, 2 stress echocardiograms, and 2 exercise-electrocardiography stress tests. Thirteen (86%) patients had CSFP in the coronary distribution implied by the noninvasive testing. Of the 11 patients who underwent previous catheterization, all 11 had prior evidence of CSFP on previous angiogram. None of these patients were on CCBs or long-acting nitroglycerin agents before angiography (Table 3). Oral CCBs were started with subsequent improvement in all 15 patients (mean follow-up time = 13.6 months). The New York Heart Association anginal class was not assessed; however, patients reported a significant reduction in the frequency of their anginal episodes. The patients were assessed on an outpatient office visit follow-up.

**Table 1.** Baseline Characteristics.

Variables	Slow-Flow (n = 15)
Demographics	
Age (mean years)	59.1
Male (%)	73.3%
Female (%)	26.7%
Comorbidities	
Hypertension (%)	86.6%
Diabetes (%)	20%
Hyperlipidemia (%)	86.6%
Body mass index (mean)	31.3
Tobacco use (%)	66.6%
No cocaine use (%)	6.6%

**Table 2.** Cardiac Findings.

Variables	Slow-Flow (n = 15)
LVEF (%)	58%
Resting ST-T EKG changes (%)	20%
ACS on presentation (%)	6.6%
CSF in LAD (%)	86.6%
CSF in the LCX (%)	20%
CSF in the RCA (%)	46.6%
CSF in 2 or more epicardial vessels (%)	46.6%

Abbreviations: LVEF, left ventricular ejection fraction; ST-T EKG, segment-T electrocardiography; ACS, acute coronary syndrome; CSF, coronary slow-flow; LAD, left anterior descending; LCX, left circumflex; RCA, right coronary artery.

**Table 3.** Medication Use Prior to Diagnosis.

Medication	Slow-Flow
Calcium channel blocker (%)	0%
Beta-blocker (%)	66.6%
Statin (%)	46.6%
Aspirin (%)	66.6%
ACE (angiotensin-converting enzyme) inhibitor (%)	26.6%
Long-acting nitroglycerin (%)	0%

## Discussion

CSFP, or cardiac syndrome Y, has distinct differences from cardiac syndrome X, one of which is that CSFP is defined by delayed opacification of contrast in the coronary vasculature during coronary angiography.^5,12^ CSFP is more often encountered in male smokers with metabolic syndrome.^13^ Our cohort is consistent with those prior reports, as 73.3% of our patients were male (mean age of 59.1 years) with 10 of the 15 patients admitting to either active or former tobacco use. The average body mass index of the cohort was 31.3 kg/m^2^, with 13 of the 15 patients having dyslipidemia.

Diagnosis of CSFP is made angiographically with demonstration of either TIMI-2 flow (ie, requiring ≥3 beats to opacify the vessel) or a corrected TIMI frame count of >27 frames, which have been proposed by Beltrame et al^2^ in addition to no angiographic lesions ≥40% and delayed distal vessel opacification in at least one epicardial vessel. Our patients were diagnosed based on the TIMI frame count and had an initial corrected TIMI frame count with a mean of 105 frames in vessels affected by slow-flow, with improvement to less than 27 frames after IC injection of nifedipine. Based on this positive IC response to nifedipine, oral CCBs were subsequently started in all 15 patients with significant symptomatic improvement in a 13.6-month follow-up.

Exclusion of alternate mechanisms of delayed coronary contrast progression is necessary to define CSFP, including coronary artery disease, coronary artery spasm, distal embolization, no-reflow as a consequence of coronary intervention, and coronary artery ectasia causing turbulent nonlaminar blood flow.^14^ Other exclusions include left ventricular myocardial dysfunction, severe hypotension, sudden increases in intracavitary pressure, valvular heart disease, air embolism, or connective tissue disorders.^6,15^

The exact etiology and pathogenesis of CSFP is not definitively established; however, microvascular dysfunction is highly suspected. Left and right ventricular myocardial biopsy specimens from patients with CSFP have demonstrated the presence of coronary microvascular disease.^6^ Small vessel disease, cell edema, capillary damage, subclinical atherosclerosis, inflammation, fibromuscular hypertrophy, and degeneration of endothelial cells with resultant microvascular luminal narrowing have been reported as existing in association with CSFP.^6,16-18^ On the molecular level, endothelin-1 and neuropeptide Y (another reason for the label “syndrome Y”) have been implicated as possible mediators of the microvascular constriction response.^6^

In contemporary clinical practice, the majority of individuals who undergo catheterization do so after an abnormal noninvasive test. Ciavolella et al^19^ reported that 69% of their 53-patient cohort with slow-flow had functional and perfusion abnormalities that matched the coronary territories that demonstrated the delayed contrast dye run-off. Similarly, we report that 13 of the 15 (86.6%) patients of our cohort had CSFP in the vascular territory affected by noninvasive testing. Hence, CSFP should be recognized as a cause of an abnormal ischemic evaluation.

On an in-depth review of our cohort’s medical record, it was noted that 11 of the 15 patients had indeed undergone previous coronary angiography. Each of these 11 patients had previously reported chest pains and had a subsequent abnormal noninvasive testing that led to the angiogram. These prior angiograms were obtained and interestingly also revealed CSFP. In fact, the most striking example were 3 patients in our cohort who underwent 3 diagnostic angiograms over a 5-year period of time. This, therefore, highlights the notion that the disease entity of CSFP is underrecognized in the community medical setting and that assigning the appropriate diagnosis may prevent additional testing.

Many pharmacologic agents have been studied in the treatment of CSFP. Studies have reported increased benefit with dipyridamole (a platelet cAMP-phosphodiesterase inhibitor) by decreasing the microvascular tone, statins via anti-inflammatory properties, angiotensin-converting enzyme inhibitors by directly modulating coronary microvascular tone, and α-blockers by decreasing sympathetic activity, thus potentially reducing microvascular tone and improving microvascular perfusion.^12,16,20-23^ Larger scale studies have not shown any real efficacy of alpha channel blocker, cAMP-phosphodiesterase inhibitors, statins, and angiotensin-converting enzyme inhibitors in improving patients’ symptoms.^12,24,25^ Nonpharmacologic methods have been reported for symptom relief in this patient population including exercise training, transcendental meditation, cognitive behavioral therapy, and transcutaneous electrical nerve stimulation.^17,26^

Of all classes of therapeutic medications that have been studied, CCBs appear to have the most efficacious role in attenuating the microvascular dysfunction associated with CSFP.^6,8,27^ A randomized double-blinded study of 80 patients by Li et al found that the oral CCB diltiazem alleviated angina, improved TIMI frame count, exercise tolerance with lessened ischemic electrocardiography response, and coronary blood flow velocity.^8^ Chang et al^11^ found that IC verapamil resulted in a significant slow-flow improvement in comparison with nitroglycerin.

A limitation to this report stems primarily from being a small study at a single center. However, this data set is from 15 consecutive patients of CSFP, and all patients were managed at the time of angiography and post-procedurally in a uniform manner. Second, there was no objective quantification such as utilization of the Seattle Angina Questionnaire to measure patients’ perceived chest pain. Third, although there was sufficient follow-up time of 13.6 months, it remains to be seen if all oral CCBs (ie, dihydropyridines, phenylalkylamines, and benzothiazepines) are efficacious for CSFP, as different CCBs have varying properties. Finally, our study surprisingly did not have any patients who were already on CCBs and no patients had repeat angiograms while taking oral CCBs to determine if they were are as efficacious as IC CCBs. This is in comparison with the study by Li et al^8^ where repeat angiograms were performed while patients were on oral CCB therapy. It can be concluded from this study, however, that IC CCB dramatically improved the angiographic finding of slow-flow and the initiation of oral CCBs in response to this angiographic finding appears promising.

## Conclusion

Coronary slow-flow should be a diagnostic consideration in patients presenting with chest pain and abnormal noninvasive ischemic testing with normal or nonobstructive epicardial vessels. Our cohort illustrates 15 patients with CSFP, of which 11 patients had previous coronary angiograms without recognition of this disease entity. In each of the 15 cases, IC nifedipine resolved the angiographic manifestation of coronary slow-flow. Furthermore, after a 13.6-month follow-up, all 15 patients improved symptomatically from their chest pain after oral CCBs were initiated.
